# Continuous quality improvement across a South Australian health service and the role it plays in a learning health system: a qualitative study

**DOI:** 10.1186/s12913-025-12557-4

**Published:** 2025-03-27

**Authors:** Mia Bierbaum, Susan Hillier, Louise A. Ellis, Robyn Clay-Williams, Angie Goodrich, Robert Padbury, Peter Hibbert

**Affiliations:** 1 IIMPACT in Health, Allied Health and Human Performance, University of South Australia, Adelaide, Australia; 2 Australian Institute of Health Innovation (AIHI), Macquarie University, Sydney, Australia; 3 Southern Adelaide Local Health Network, Adelaide, Australia

**Keywords:** Quality improvement, Continuous quality improvement, Implementation science, Capacity building, Qualitative research, Ambulance ramping, Patient flow, Learning health system, Consolidated framework for implementation research, Thematic analysis

## Abstract

**Introduction:**

Continuous quality improvement (CQI) initiatives are commonly used to enhance patient safety and quality of care. A novel South Australian Local Health Network (SALHN) Continuous Improvement Program (CIP009) has integrated a top-down model of executive-directed change initiatives, with a bottom-up approach of clinician designed interventions to address an organisational-wide goal of improved patient flow. This study evaluated the strengths and challenges of CIP009 implementation from the perspective of participants and deliverers.

**Methods:**

A qualitative study was conducted in 2023/2024 to evaluate the implementation of CIP009 and 12 associated quality improvement projects. Semi-structured interviews and focus groups were conducted with key stakeholders (executives, coaches and CIP009 fellows) and guided by the Consolidated Framework for Implementation Research (CFIR). A document review and observations of CIP009 team meetings were also conducted. Data were analysed inductively using thematic analysis, then deductively mapped against the five CFIR domains.

**Results:**

Thirty-one participants were interviewed individually or in focus groups, two presentation days and six team meetings were observed, and 78 documents were reviewed. Seven key themes were identified highlighting key challenges and strengths of CIP009 implementation within the SALHN setting. These included four key strengths:* the CIP framework and culture *(the flexible framework, common language, training, and a culture of flattened hierarchy); *the benefits of support from a dedicated, internal improvement Faculty* (wrap around support from coaches); *the advantages of an enthusiastic team member disposition and incentives *(vested interests to enhance workflow and patient outcomes); and e*ffective teams and team composition *(teams comprised of senior clinician change agents). Three key challenges included: *workforce and organisation-level challenges *(individual workloads, workforce capacity, and data access); *team cohesion, logistics and stakeholder engagement challenges *(issues in the way teams worked together); and *training and support shortcomings* (the training course, and the top-down nature of CIP009).

**Conclusion:**

This evaluation identified that CIP009 was considered an effective multifaceted CQI program. The strengths of CIP009 support a learning health system (a data driven model, utilising systematic frameworks, with commitment from leadership, and a culture of continuous learning). Further integration of implementation science principles may support the program to overcome the key challenges identified. These findings will inform and guide improvement efforts within future iterations of CIP.

## Introduction

Continuous Quality Improvement (CQI) capacity and capability building are important and widely used methods [1–4] to improve care pathways and service delivery in healthcare organisations [5, 6], and increase patient safety [7]. This is achieved by identifying, analysing and addressing quality issues and enhancing the efficiency of resource allocation [3, 5, 8]. It requires affective commitment from staff who identify a need for change as well as strong leadership support and active engagement [9]. Capacity building ensures there are enough staff trained in QI methods to implement projects, while capability building develops staff skills and confidence to implement QI projects [10].

The implementation of individual [11, 12] or organisation-wide projects [13–17] are well documented in the literature. Examples include the Interprofessional QI program in the Netherlands, which facilitated interprofessional healthcare teams to design QI projects following online training and continuous support [16]; and the Safer Patients Initiatives in the U.K. which were whole-of-hospital, pre-prescribed (top-down) clinical improvements that were locally adapted [15, 17]. There has been limited examination of the barriers and facilitators to effective implementation of sustainable QI training programs [5], in particular cross disciplinary whole-of-hospital programs to improve quality of care through a combined top-down and bottom-up approach, warranting further investigation.

The Continuous Improvement Program (CIP) has been run for 20 years by the Southern Adelaide Local Health Network (SALHN), in South Australia. SALHN encompasses a tertiary teaching hospital, and a regional community hospital, as well as sub-acute, mental health and primary care services, with approximately 700 acute hospital beds [5]. Early iterations of the CIP were developed by the SALHN Department of Surgery and Perioperative Medicine in 2004 [5]. CIP was adapted from frameworks [18] including Lean methods and process redesign principals [19, 20], Model for Improvement methods [13], and key learnings from Intermountain Healthcare, Utah, USA [21], to suit local needs [22].

An earlier iteration of CIP that focused on building capability across the workforce, was evaluated [5]. This identified that CIP is led by an internal Continuous Improvement Unit (the Faculty, comprised of QI specialists with predominantly clinical backgrounds) who support and mentor staff to enhance their QI skills and knowledge, and facilitate local CQI projects [5]. The CIP training is conducted with staff across the service and is designed to teach them how to identify issues in the workplace, to problem solve and implement sustainable solutions by systematically using the SALHN 8-step continuous improvement framework [5]. Projects are designed and implemented by frontline healthcare workers at the interface of patient care, with the aim of achieving buy-in and adoption from healthcare staff. Project teams are trained and supported through continuous coaching from the Faculty to redesign processes, maximise capacity, enhance efficiency and reduce waste; all key strategies in overcapacity management [23]. Teams are supported to access data to measure baseline processes and monitor improvements, as well as provided with overt organisational permission and executive support for the interventions [5].

The most recent iteration of the program, CIP009 (2023/2024), is a novel CQI program which has been conducted using an innovative combined top-down and bottom-up approach. This integrates executive codesign of 12 CIP009 intervention topics aligned to hospital strategic priorities, with clinician design and implementation of 12 associated microsystem CQI projects. CIP009 has an overarching strategic macro-objective driving the projects to increase improvement capacity and capability and reduce ambulance ramping across SALHN hospitals. Emergency Department (ED) congestion and ambulance ramping is a persistent challenge, whereby the handover of patients from paramedics to ED clinicians [24] is delayed when patient flow from the ED across the hospital is impeded by various bottlenecks [24], and demand and bed capacity mismatches [23]. Ambulance ramping has been shown to result in delayed triage and care, increased length of stay (LOS) and rates of admission, in addition to workforce burden and stress [24]. While ramping is related to increased demand for ED services and staff shortages across ED and ambulance services, challenges associated with hospital-wide patient flow also contribute to these issues by delaying patient transfer out of ED creating further delays for proceeding ED patients [24]. This qualitative study aimed to: 1) characterise the SALHN CIP009, a long-term improvement capacity and capability building training program, to understand program processes and context and; 2) examine the strengths and challenges of implementing 12 clinical micro-improvement projects as perceived by CIP009 team members, coaches and executive involved.

## Methods

### Study design and setting

An exploratory, inductive and deductive qualitative study design [25] was used to evaluate the SALHN CIP009 and to characterise the program. Interviews and focus groups were conducted with executives affiliated with CIP009, CIP009 coaches from the CIP Faculty, and individuals who participated in CIP009 training and projects, referred to as CIP009 fellows (typically doctors, nurses, and allied health professionals, such as physiotherapists). Observations of presentations and training sessions, and project team meetings were conducted, and documents were reviewed to characterise the program. The study design, analysis and findings are reported in line with the Consolidated Criteria for Reporting Qualitative Studies (COREQ) [26]. Human Research Ethics Committee (HREC) and governance approval for Low and Negligible Research (LNR) by the SALHN HREC (LNR Reference number: LNR/23/SAC/157.23; and Office for Research: OFR Number: 157.23) was obtained before research commenced.

### Recruitment and sampling strategy

Recruitment of CIP009 fellows, coaches, and executives was conducted by key contacts from the Faculty, via email or in person in late 2023/ early 2024. This included an invitation to participate in interviews, focus groups or observations, followed by reminder emails. The Faculty recruited fellows from the 12 CIP009 project teams. Each team had varying numbers of team members, typically four per core team, plus one or two coaches per team from a pool of 9 coaches. The Faculty also recruited executive staff aligned with the CIP009 projects. The total number of CIP009 fellows and executive approached is unknown. Participation was voluntary, and responses were treated confidentially with data de-identified. Purposive sampling [27] was used to ensure inclusion of participants from a range of health disciplines and with varying levels of experiences, and participants self-selected by responding to recruitment invitations. All participants provided written informed consent before participating in the study, including an interview, focus group, being present during an observed meeting, and approving review of program documents.

### Data collection

Interviews, focus groups, document review and observations were conducted in parallel between October 2023 and February 2024. The semi-structured interview/focus group topic guide was used to elucidate perceived strengths and challenges of the CIP009 (Table 4 in Appendix). Questions were developed and reviewed by the research team and informed by the domains of the Consolidated Framework for Implementation Research (CFIR) [28]. All interviews and focus groups were audio-recorded, transcribed verbatim, and deidentified. All data including recordings of interviews/focus groups, observations and presentations, as well as other program documents, were stored on a secure password protected University server. Interviews were conducted by the first author (MB), an experienced qualitative researcher (PhD) who had no preexisting relationship with participants. Only the interviewer and interviewee(s) were present, and interviews were typically conducted on site in an office at a SALHN hospital, over the phone or by videoconference. Interview and focus group recruitment continued, with iterative analysis until data saturation was reached, and no new themes emerged [29].

Observations of CIP009 midpoint ‘report back’ and graduation sessions (where teams present their project progress and receive certification for completing the training course), as well as project team meetings were conducted in real-time or via video recording. All non-participatory observations were conducted by MB, and field notes were taken about communication and interactions between team members, as well as CIP009 processes and strengths and challenges to implementing the projects. Project team members were made up of CIP009 fellows, coaches and core stakeholders. This approach [30] was used to observe team interactions and communication, how project work was planned and conducted to develop a deeper understanding of the program. MB reviewed CIP009 documents, such as training slides and notes, midpoint and graduation presentation slides, team meeting minutes, and support resources, to develop a clearer understanding of the program processes and content.

### Data analysis

Iterative and inductive thematic analysis [31] was used to analyse the data from the interview and focus group transcripts, and observations field notes [32]. MB conducted the initial coding of transcripts, by reviewing the transcripts twice for familiarisation, then coding line by line to identify key codes and potential themes [32], using the NVivo software v.14 [33]. Once an initial coding framework was developed, MB recoded the data to verify the initial framework. Senior author (PH, professor) then reviewed the coding of a 10% sample of transcripts, after which themes and codes were discussed and refined, and disagreements resolved through team consensus decision making. Once the coding framework was finalised, MB recoded all the transcripts for a third time and finalised the key themes. Exemplar quotes were chosen to support the thematic framework, within which participants are identified by a code to maintain confidentiality. The document review and analysis of observation notes informed the characterisation of the CIP009 program including mapping the milestones of the program, roles of participants, and key strengths and challenges in line with the key themes identified.

A deductive analysis of data was then conducted by MB to map the themes, subthemes and codes across the five CFIR domains [28]. The CFIR is a widely used framework for assessing implementation evaluation [34] and was used to both inform the data collection, and to reassess the challenges and facilitators of the CIP009 implementation within SALHN. Data were triangulated to corroborate the findings across the three methods of data collection (interviews/focus groups, observations and document review) and across participants from different roles and backgrounds (executives, CIP coaches and CIP009 fellows) [35]. Member checking was utilised through return of transcripts to interview participants to validate or amend the content before analysis began [35]. These techniques were used to enhance the reliability of the data analysis [35].

## Results

Between October 2023 and February 2024, 31 participants (including CIP009 fellows, coaches and executives) were interviewed individually (across 27 interviews) and/or in three focus groups either face-to-face, over the phone or by video conference with the lead author (Table 1). Each of the 27 individual interviews were on average 28 min long. Three focus groups were conducted by MB using the same questions with: CIP009 fellows (*n* = 2 participants, 20 min); coaches (*n* = 6 participants, 80 min); and executives (*n* = 2 participants, 18 min).

**Table 1:** Interview/focus group participant demographics

Interview/focus group cohorts		*N*
CIP009 executives		6
CIP009 coaches		9
CIP009 fellows		16
CIP009 fellow clinical experience (Range 2–40 years)*	10 years or less	2
	11–20 years	6
	21 years or more	4
CIP009 fellow Profession	Nurse	5
	Doctor	7
	Allied Health Professional	4

*4 CIP009 fellows did not report their length of clinical experience

In parallel to the interviews and focus groups, five CIP009 team meetings were observed, typically comprised of five team members including CIP009 fellows, stakeholders (such as clinicians for whom the improvement will impact), and a CIP009 coach, each running for an hour on average. One Faculty team meeting was observed (*n* = 9 participants, 60 min). Field notes of the observations documented that teams typically discussed project progress, challenges, and made action plans for next steps. The midpoint presentations were observed (4.5 h), as well as the graduation session (4.5 h) and field notes were taken. Each team presented their progress at each of these sessions. CIP009 Faculty and team documents were reviewed (*n* = 78 documents), such as: the training agenda, slides and notes, and recordings of guest lectures, midpoint and graduation presentation slides, recruitment and registration documents, support resources, team meeting agendas and minutes, project plans, project specific outlines of length of stay data, and draft protocols, training and presentation evaluation data. Analysis of these data enabled the characterisation of the program, along with the identification of key strengths and challenges associated with CIP009.

### Characterisation of the SALHN CIP009 program

#### CIP

The document review, observations, interviews and focus groups provided data to characterise the CIP009 program and provide context for the evaluation. Since 2018, nine iterations of the CIP have been delivered, supporting over two hundred internal CQI projects over that timeframe. This has increased organisational awareness of the program, approaching a critical mass of staff having graduated from past CIPs, or with experience as CIP project stakeholders. The CIP is historically a 6-month CQI program delivered to staff which includes training sessions around the SALHN 8 step methodology [5], and continuous support from CIP coaches and Faculty. CIP009 fellows present project progress to their cohort at a midpoint presentation and at the graduation session (Table 2).

#### CIP009 design

In preparation for CIP009, 12 CIP project topics were selected and codesigned by hospital Division and CIP executives based on metrics such as high rates of admission, readmission, or length of stay. Project topics were designed to include at least two hospital Divisions involved in the patient care continuum, facilitating collaboration across the organisation. The CIP Faculty then conducted preliminary data analysis of the projects to gather baseline data and background information to justify and prepare each project for the 12 teams (Table 2).

#### CIP009 recruitment

CIP009 fellows were typically nominated by their Heads of Units and Divisions and assigned to a CIP009 team. CIP009 fellows (doctors, nurses and allied health professionals) were multidisciplinary, with varying levels of seniority, from multiple divisions across SALHN. Each team was led by at least one CIP coach and some with an additional shadow coach in training. Team members were introduced to their coach by the Director of CIP and presented with the preliminary analysis and justification for the project topics (Table 2).

#### CIP009 training and support

CIP009 fellows were provided with 3.5 days of training about CQI methodology and key objectives of CIP009 (Table 2). Fellows were provided with resources to support the development of these skills. Sessions were delivered as seminars by the Faculty and senior executives, including shared experience of past CIP projects, and group workshops focused on practical cases. During and following the training sessions, teams initiated the CIP 8-step continuous improvement process, to identify, define and address their project issue. The most commonly reported data collection methods teams utilised included audits, electronic medical record analysis, observations and staff and patient surveys. The projects aimed to increase patient flow across micro-systems, with the intention of improving hospital-wide patient flow through the reduction in patient admission, readmission, length of stay and unwarranted clinical variation.

The CIP009 teams were guided by improvement coaches, and the Faculty. Coaches played a project management role, accessing, conducting and supporting data analysis, providing expert CQI advice, and developing outputs such as presentations and protocols through face-to-face and virtual support. CIP009 teams met with their coaches regularly to discuss the project design and implementation plan. Coaches had a range of clinical backgrounds and CQI experience. All were graduates of a past CIP course and had shadowed another coach supporting a previous CIP team. Coaches received in-house training and mentoring and regularly collaborated in Faculty brainstorming sessions to discuss CIP009 projects.

In light of the complexity of projects, the Faculty and coaching support provided to CIP009 project teams was extended from a six-month program to over 18-months to enable teams to complete the SALHN 8 steps with wraparound support (Table 2). As a result, at the time of the evaluation, teams were still in the diagnostic and planning phases and had not completed the SALHN 8 steps. Teams had typically refined the problem, conducted analysis including development of a cause-and-effect diagram, and identified outcomes to be measured.

**Table 2:** Key CIP009 project milestones

**CIP009 Project Milestones:**	
***Preparation:***	
• **CIP009 projects (*n*** **= 12) were codesigned** with clinical directors and SALHN division leadership	
** Team**	**Topic**
1	Shorter Stays, Better Journeys: Improving back pain care
2	Alcohol presentations to ED
3	Preventing Delirium on 4D
4	Not just a failing heart, Standardising Heart Failure Presentations
5	Standardising SALHN Mental Health Care Pathways for Clinical Presentations of Borderline Personality Disorder
6	Reducing short stay Undifferentiated abdominal pain admissions
7	Improving the patient flow processes at Southern Adelaide Palliative Care Services
8	Toe-Tal Improvement: Ramping Up Care for Patients with Diabetic Foot Infection
9	Future of Falls in Elderly at FMC
10	Bringing AIR (Acute Illnesses of the Respiratory Tract/System) in and out of Flinders Paediatrics
11	ED to Emergency Extended Care Unit Pathway
12	PV bleeding presentations to ED
• CIP009 fellows were nominated by division executives	
• Pre-training data analysis and project preparation conducted by coaches to justify projects to teams	
• Team introductions by director of CIP, preliminary analysis of projects discussed	
***Commencement of CIP009*:**	
• **CIP training days** (March 2023) including project team groupwork with coaches.	
Training day (hours)	Topics
Training day 1 (4.5 h)	Introduction to the ‘Towards Zero Ramping: Improving organisational capability through standardisation’ International Guest lectures-A Personal Journey in Acute Care Improvement
Training day 2 (8.5 h)	Welcome from the Minister for Health and wellbeing Standardisation in clinical practice – reducing unnecessary variation Project pathways-Introduction of teams and project streams Continuous Improvement Principles Group Activity Continuous Improvement Program – 8 step Improvement Framework Diagnostic Tools (1) Breaking down the problem, focus on process mapping, tracking Work as imagined, work as done Allocation of small groups Part 1: draft milestones & stakeholders for process map (breakout rooms)
Training day 3 (8.5 h)	Human Factors – The Influence on Healthcare Quality Measuring for Improvement Lessons learnt from protocol development over 20 years Small Group Work Part 2: further analysis of pathway and identify key steps/milestones (breakout rooms) Asking why Diagnostic Tools (2) Understand what to work on (tally sheets, brainstorming, Ishikawa, multi voting, Pareto charts) Consumer involvement- The value of having consumers on projects Small Group Work Part 3: discuss diagnostic plan (direct observations, plan mapping meeting, measure & mission statement)
Training day 4 (7.5 h)	Data Clinician interface Ethics Approval Group Work – Part 3 continued: diagnostics: what will you do tomorrow? How to Publish Your Project Evidence: SALHN CI Sustainability Plan your Work, Work your Plan! Ready to Launch
• Teams initiated the **SALHN 8 step continuous improvement framework** process	
** Step**	**Task**
1	Define the Problem
2	Breakdown the Problem
3	Set a Target/Mission Statement
4	Root Cause Analysis
5	Improvement planning
6	Implementation
7	Evaluate/Assess Impact
8	Continuous Improvement
• **Continuous Support**: CIP009 Project teams were provided with continuous support from coaches and Faculty (approximately 4 h of support each week per team, via team meetings, data collection and analysis)	
• **Stakeholder Engagement** To further elucidate the root causes of selected problems, CIP009 fellows recruited stakeholders to provide clinical insight and local knowledge to the problem-solving process through brainstorming and process mapping	
• **Midpoint Report back session** (June 2023) (4.5 h) The project teams presented their progress at a midpoint report back session, and received feedback from the CIP009 teams, coaches, and Faculty. The hospital CEO and other SALHN executive attended these sessions and provided feedback to teams	
• **Graduation Report back session** (October 2023) (4.5 h) The teams presented their progress to their CIP009 peers, coaches, Faculty and executive at their graduation ceremony, demonstrating their use of the 8 step CIP framework to design and implement a service improvement. Most teams had not completed the 8 steps by this point. They had refined the problem, conducted analysis including development of a cause-and-effect diagram, and identified outcomes to be measured	
• **Planned ‘Where are you now?’ report back session (October 2024)** The teams will present their progress and receive feedback from peers and executive.	
• **Planned Sustainability following graduation** (continuous support from the Faculty anticipated until project completion in June 2024) At intervention stage, teams report progress to executives and consumer adviser committees, for accountability and sustainability	

Thematic analysis of data identified seven key themes highlighting key challenges and strengths of CIP009 implementation within the SALHN setting: Four of the themes were focused on strengths of CIP009 implementation and captured concepts like: flattened hierarchy; wrap-around support from coaches; vested interests; and senior clinical change agents. Three themes were focused on key challenges of CIP009 implementation and included: individual workloads; issues in the way teams worked together; and training shortcomings. Exemplar quotes are presented.

#### CIP009 strengths

Overwhelmingly, CIP009 fellows, coaches and executive were positive about CIP009, and the improvements achieved by the teams. Four themes and subthemes were identified as strengths of CIP009 that facilitated the implementation of the projects. Exemplar quotes are presented throughout the results.

#### Theme 1: CIP framework and culture embedded in the psyche of the SALHN organisation

##### A flexible and adaptive evidence-based program

Key strengths of CIP009 included the flexible, adaptive, agile and transferable nature of the CIP methodology and the predetermined and clear nature of the projects. This enabled coaches to do preparation work identifying key literature and baseline data to present to teams and facilitated efficient problem definition and change implementation. The report back presentation sessions at midpoint and graduation were seen as an opportunity to learn from other teams and celebrate successes. Presentation deadlines held teams accountable, and the extended timeframe of CIP009 support facilitated progress of projects. CIP009 fellows valued the protected time for training, away from clinical duties, to immerse themselves in the CQI topics. Many felt additional protected time would accelerate project progress.

*“That’s a really valuable thing for a clinical leader to be taken out of the environments [so] that they can just really focus on [CIP009].” (p31*,* CIP009 fellow)*

##### Professional relationships, buy-in and engagement

Achieving stakeholder buy-in and project engagement was considered essential to change, facilitated through coach support and networking. CIP009 fellows valued the multidisciplinary and cross-divisional collaboration (particularly with ED), facilitated by CIP009, both in the composition of the teams, and engagement with stakeholders during brainstorming sessions and protocol development. This enabled teams to develop a clearer understanding of the patient journey end-to-end and strengthen professional networks. Consumer involvement in projects was considered important but only utilised across some projects.

*“One of the key*,* kind of*,* crucial*,* it was the culture piece as well*,* to say ‘Actually this is what’s happening in my piece of the world. But what’s happening over there in yours?’ And that has been probably one of the biggest things when I’ve gone to a lot of the process mapping etcetera*,* it’s just the team seeing an alternate view or alternate perspective of how that patient is managed.” (p6*,* executive)*

##### Awareness of CIP and a culture of enquiry

SALHN was perceived to be moving towards critical mass regarding CIP training saturation, with awareness and engagement with CIP increasing exponentially. CIP has built a culture of inquiry over time, across SALHN, with continuous improvement ideas perceived to be embedded in the organisational psyche. The use of a standardised, adaptive and evidence-based framework to develop targeted improvements, that is simple to follow and adapt to local problems, was valued.

*“We’ve had nine other CIPs where we’ve trained a lot of other people*,* like*,* I think in terms of the trust and the interest and the knowledge of the general workforce in terms of even just participating in mapping sessions*,* I do think that’s been a critical factor to the success of this one [CIP009]*,* in the sense of*,* you know*,* people trusting the [CIP] process.” (p20*,* CIP009 coach)*

##### A strategic approach to capacity and capability building

The SALHN CIP training builds improvement capacity and capability by teaching fellows the skills to independently design and implement improvement projects. CIP009 however, had an outcome-focused strategic direction imposed upon the projects, with greater coaching support provided to facilitate and expedite progress of improvement projects. CIP009 was focused on organisational capacity building, efficiency and reducing waste, built on the foundation of organisation-wide CIP awareness and use of a common CQI language. This facilitated engagement with stakeholders who were already familiar with CIP. CIP009 fellows valued the overarching hospital priority-aligned strategic approach used to address network-wide wicked problems (Complex, unpredictable, challenging and intractable problems [36]) and the non-prescriptive combined top-down and bottom-up nature of CIP009.

*“[We used] the CIP as a strategic plan to be able to look at involving clinicians at the patient-clinician interface to systematically fix ambulance ramping because we know that ambulance ramping is a symptom of delays across the entire quantum of care.” (p6*,* executive)*

##### Accountability

While executives nominated and codesigned the broad selection of CIP009 topics, clinicians at the patient interface valued their ownership over the design and implementation of the improvement projects. CIP has established avenues for ongoing accountability and sustainability of the improvement projects through regular reporting to committees and executives.

*“Everybody jumped on board because we all had a common purpose*,* so that was fine. But I think the real strength of it is that you can*,* you know*,* yes*,* you may well be given an area*,* but you can really delve down what’s most important and really focus on that.” (p19*,* CIP009 fellow)*

##### A culture of flattened hierarchy

The CIP Faculty and program instilled a culture of flattened hierarchy, enabling CIP009 fellows to confidently engage with and discuss ideas across the team, enhancing collaboration. This was established through role modelling with coaches demonstrating humble enquiry and negotiation techniques as methods to constructively challenge current practices, and support change adoption.

*“[The CIP Faculty are] very good at*,* I think*,* challenging the way that some of the ED people think*,* and actually in reshaping that. But also*,* I guess empowering them to say what’s wrong and involving them in the process of improving it. Umm. So yeah*,* we love the CIP.” (p22*,* executive)*

##### Training strengths

The CIP009 training sessions held off site were considered well-structured with interesting in-depth content. The theory and reference to the literature throughout the training content was generally well regarded, and participants felt the framework was applicable across disciplines. Many CIP009 fellows valued the lectures from the expert presenters (including international guests), the small group activities and the real-life examples of past CIP projects presented by alumni and coaches. These examples of learned experience, alongside the SALHN 8 step framework, were useful to shift mindsets around continuous improvement methodology. The team-building benefits of the face-to-face sessions, and opportunities to network with other teams, coaches and CIP009 fellows, as well as the provision of training resources were also valued.

*“I think the fact that the facilitators were able to relate past stories or past examples where the [CIP] process had worked*,* it was really good. So*,* we knew that even if we were early on in the process and it wasn’t*,* and it wasn’t really clear what direction we were heading*,* we knew that we have people who were experienced in this*,* had gotten results and the process had worked for them. That was a key motivator throughout.” (p2*,* CIP009 fellow)*

#### Theme 2: the benefits of support from a dedicated, internal improvement faculty

##### An experienced internal faculty

Participants were complimentary about the large and experienced Faculty and leadership supporting the 12 CIP009 projects, and the breadth of knowledge coaches demonstrated. CIP Faculty executives played a key role as gatekeepers of coach workload to protect coach capacity to support CIP009 teams. The increased provision of coach support for CIP009, relative to past CIPs, resulted in a perceived higher standard of project outcomes.

*“They’re very experienced and they can see the wood for the trees*,* and I think that’s really valuable.” (p23*,* executive)*

##### Stable continuous support from an internal and well-resourced faculty

The continuous and resourced nature of Faculty CIP009 support was invaluable and seen to minimise the workload burden on CIP009 fellows and ensure projects progressed. The internal nature of the Faculty meant the coaches could provide indispensable organisational knowledge-based advice. The Faculty also advocated for improvement changes that required policy escalation or changes to workflow and helped to navigate occasional challenging dynamics across divisions, as neutral stakeholders.

*“We have that capability that’s in-house*,* we can network well with the process owners*,* and we can leverage that in a*,* in a very*,* very critical manner*,* comparative to other organisations. So*,* people who in another situation*,* in comparison with other organisations*,* consultants would come from outside organisations like [consulting firm names]. They would come*,* recommend and go*,* but they would not stay for the whole process. But I think we have from start to finish*,* end to end visibility*,* engagement.” (p16*,* CIP009 coach)*

##### Clinical directors and coaches embedded in divisions and within executive structures

Faculty staff who were embedded within executive teams and divisions, wielded influence to engage executives with change initiatives. High executive and leadership awareness, understanding and support of the CIP009 projects across SALHN was perceived to facilitate improvements, staff buy in, and minimise governance barriers. Participants also felt that executive attendance at the CIP009 training and presentation days increased recognition of and institutional support for their improvement initiatives. Similarly, ED leadership support of projects validated improvement programs and facilitated staff buy-in.

The coaches who were embedded in Divisions were considered particularly helpful as they had pre-established relationships with staff, facilitating stakeholder engagement with the projects, as well as having greater clinical understanding of the project. Coaches were considered experts in improvement, with their process knowledge helpful to guide feasible intervention design and facilitate change. Coaches were also seen to have strong professional networks which were useful to progress interventions.

##### Continuous wraparound support from knowledgeable and passionate coaches

Coaches and Faculty staff were considered to be a key strength of the CIP009 program demonstrating enthusiasm, commitment and belief in the value of each project. Coach support was respectful and encouraging, but not prescriptive. Coach clinical knowledge was another key strength perceived to facilitate project progress. Coaches aimed to provide a standardised approach to project support and facilitation, and the Faculty team promoted a culture of support and beneficence through their training, resources and coaching, which facilitated engagement with stakeholders.

*“We live and breathe this*,* and we I think every single one of us in this room 100% believes in the [CIP] methodology. And we have a point to make now that this methodology can make a difference.” (p14*,* CIP009 coach)*

The extensive wrap-around support from coaches who were embedded in teams was considered a key strength of CIP009. This included data sourcing and analysis, proactive project management, and output development such as protocols and preparation of presentations. This reduced the burden on CIP009 fellows and freed up time to provide expert clinical advice on the improvement project design and implementation. The coaches led the teams through the 8-step continuous improvement framework, providing structured guidance and feedback and preventing teams from jumping to solutions.

*“The CIP team as a whole have been an amazing support for the ED this year*,* but they are very good at doing a wraparound support*,* I guess to take some of the smaller tasks away from us*,* you know*,* data collection. They’re very good at presenting the data analysis*,* and I think in trying to change the way that you think. I think as clinicians*,* we are good at jumping at problems and solutions very quickly. And I think*,* in slowing down that process*,* sometimes you really get the data you need to really understand the problem*,* which I think is really valuable.” (p22*,* executive)*

##### Regular multimodal meetings with coaches, and clear respectful communication

Coaches coordinated regular meetings and communication between team members to maintain project momentum and hold team members accountable, without overburdening them. CIP009 fellows valued these often-weekly meetings, particularly the flexible nature of the hybrid face-to-face and virtual meetings, and clear communication about expectations, task setting and virtual communication when they were unable to attend in real time.

#### Theme 3: the advantages of an enthusiastic disposition and incentives

##### CIP009 fellow disposition, belief in the program and skill level

Individual CIP009 fellows’ disposition was considered to have an impact on project progress, with an appetite for change, and respect and belief in CIP009 to achieve change being valuable characteristics. Fellows, naturally, began the CIP009 course with varying skills and experience, but their capacity to be open to feedback and to show initiative was beneficial. The CIP009 process helped fellows gain insight into the contributing factors of their project problem, which were often different to what they expected. Project progress was best supported by fellows who managed their time to complete project tasks and meet with their teams regularly, by prioritising other work commitments. Past CIP alumni had often become continuous improvement advocates themselves after graduating from CIP.

*“A lot [of CIP fellows] then go on to really become fierce advocates and do continue to do things because it becomes*,* they adopt this*,* this*,* as their way of doing business. And that really does assist in reaching a tipping point within the organisation of enough people to really do things at scale… one of the greatest things to initiate cultural change is to align people on an improvement journey.” (p17*,* executive)*

##### Incentives, a shared vision of beneficence and improving workflow and patient care

There were various incentives identified to complete the CIP009 project including: a shared vision of beneficence and developing capability to improve patient support end-to-end; benefits to career progression; continuing professional development (CPD) points; easing workflow demands for staff; learning how to break down problems and design and implement effective feasible solutions; opportunities to network and collaborate with consultants to improve processes; gaining new perspectives on patient journeys from team members; supporting teammates; and opportunities for publication. Almost all participants reported a vested interest in the improvement being delivered effectively, with many projects being seen as impactful and meaningful to the CIP fellows.

#### Theme 4: effective teams and team composition

##### Multidisciplinary teams that balance expertise and capacity to enact change

Team cohesion and collaboration in the teams were important factors, ensuring that fellows felt solutions to the identified problems were not imposed upon them, but generated together. The composition of team members was important, with value seen in having a balance between expertise from more senior medical staff, and members with capacity to *do* the work, with the later role predominantly falling to nursing and allied health-based team members. However, these staff often reported not having time to ‘do’ the work on top of their clinical workloads.

*“I think the strengths are the level of expertise of the people that are participating. The fact that it has support from the CEO here at [hospital]*,* and you know high levels here at [hospital]*,* it’s definitely a priority that we’re all interested in working towards*,* and people are very motivated to make change in that area. Especially people who have come on board from general medicine.” (p1*,* CIP009 fellow)*

##### Senior change agents and engagement with stakeholders

The more senior CIP009 fellows were seen as change agents who facilitate change adoption, particularly through medical and surgical staff engagement. The multidisciplinary nature of teams was seen as a strength of CIP009. Familiarity with team members was also considered valuable, with pre-existing rapport facilitating smoother teamwork. Ensuring the team members were engaged and positive about the project was important, as was engaging the right stakeholders, particularly those from ED, to provide input and new perspectives.

#### CIP009 challenges

This iteration of the CIP program had a focus on improving patient flow in comparison to past capability building CIPs. CIP is firmly embedded within SALHN culture, with CIP language common across SALHN, and leadership support facilitated through executive, and senior staff involvement in the CIP training and projects. Despite these factors, challenges persist. The thematic analysis identified three themes that represented challenges of CIP009. Exemplar quotes are presented throughout the results.

#### Theme 5: workforce and organisation-level challenges of improvements

##### Clinician workloads, competing priorities and time

Limited time and capacity to engage in the project was the most commonly reported organisational-level challenge for CIP009 teams. Competing priorities and clinical duties limited opportunities to meet and coproduce the work. Some felt that the timing of their improvement project implementation was impeded by other priorities that detracted from stakeholder engagement with the projects, such as accreditation. Participants talked about the importance of teams being ready, mature and capable for CIP009 and how if the team was in crisis-mode, from other stressors like workforce issues or seasonal demand, this was seen to detract from their ability to conduct improvement projects effectively.

*“We’ve got a capability level that doesn’t match what the CIP was trying to pull us to. [Our] NUM [Nurse Unit Manager] is absolutely stretched beyond capacity… Does she have time to do this other extra thing? No… We did feel a little bit like somehow this process was generating pressure and it was generating pressure in a way that wasn’t always helpful.” (p29*,* CIP009 fellow)*

##### Workforce capacity

Workforce capacity and operational demand challenges included balancing annual leave, staff capacity with seasonal fluctuations in operational demand, and workforce shortages. Fitting the additional workload of CIP009 into daily workflow was challenging for many and created additional pressure. This was alleviated to some extent by the extensive wraparound support provided by coaches. Many CIP009 fellows noted that there was no sanctioned time to engage with the projects, other than the training days, mid-point and graduation sessions. They posited that additional protected time from clinical duties to immerse themselves in the project would facilitate the implementation of each improvement project. The timeframe of CIP009 (despite the extension) was perceived by many as too brief to progress through the SALHN 8 steps and achieve the types of improvements that had been designed, increasing pressure on fellows.

*“My personal view is that [6 months] is too*,* too quick to*,* you know*,* and we did spread it to what it ended up being [many more] months. And I*,* my personal view is that*,* you know*,* at least a nine-month course would actually give that time… But I think that*,* you know*,* six months*,* like*,* with sick leave and people’s annual leave*,* and you know*,* so it ends up not there in six months if people take some leave in between.” (p9*,* CIP009 coach)*

##### Data access and quality and infrastructural challenges

Another frequently discussed challenge was the poor access to electronic patient data and poor data quality (due to documentation variation) to support the improvement process. Access to data for both baseline problem analysis, and monitoring of change was a challenge noted by many participants, and led to project delays, frustration, and increased workload for the coaches. Delays to technological infrastructure (ICT) improvement changes, limited physical infrastructure such as bed capacity for improvement projects, governance approval processes delays, and medico legal barriers (which were reportedly time consuming to navigate), were all thought to impede project progress.

*“That’s the other challenge is when we come up with some interventions and it’s anything to do with [the electronic medical record*,* EMR]. It’s a statewide EMR system. So*,* we need to make sure that every other [Local health network*,* LHN] providing the same service actually want to invest in that as well. So*,* we’ll put an improvement ticket in*,* but it takes years for anything to happen. So*,* that’s probably another challenge and a barrier to implementation.” (p8*,* CIP009 coach)*

#### Theme 6: team cohesion, logistics and stakeholder engagement challenges

##### Team logistical challenges

Team-based challenges were predominantly around logistics with team members and stakeholders located across divisions and locations making it challenging to schedule project meetings. This resulted in poor momentum for some teams. Participants felt that the composition of their core teams could have had greater representation from different Divisions, specifically ED, and General Medicine. Participants posited that greater involvement of diverse stakeholders, especially those previously CIP trained, would have enabled a greater understanding of the improvement projects, and enhanced adoption of the changes.

“*Initially in our CIP*,* we did not have the emergency physicians… And not having any representation from emergency was a bit hard.” (p10*,* CIP009 fellow)*

Several CIP009 fellows and coaches discussed how challenging it was when there was unequal contribution, engagement and collaboration from team members. The composition of CIP009 teams was purposefully skewed toward more senior, executive, medical and surgical-specific staff who were perceived to be more time poor than their nursing and allied health counterparts. Utilisation of these individuals’ expertise and seniority meant that there was a greater reliance on coaches to provide the wrap around support.

##### Team cohesion challenges

Some CIP009 fellows discussed poor team cohesion and a lack of consensus to be a challenge to overcome as they progressed, particularly when the team lacked clarity around the definition of the problem they were provided with. Project complexity, including complex patient cohorts, made problem definition challenging, impacting the design and implementation of feasible improvements. Similarly, not having a prior relationship with their team members meant some felt less accountable to their team. Careful team and coach alignment, as well as trust and rapport between teams and the Faculty were important to ensure fellows felt confident they would be supported to succeed.

*“I didn’t know the team. Yeah*,* like*,* we were all strangers… When you don’t have a personal relationship with someone in the team*,* you don’t feel as accountable to them… If I’m working with my colleagues*,* they’re my friends. Like*,* you don’t want to let them down… I think it was tricky trying to work with people that you’ve never worked with before.” (p30*,* CIP009 fellow)*

##### Lack of engagement and buy-in

Lack of engagement from stakeholders across the hospital (particularly surgical and medical-based clinicians and ED stakeholders) and resistance to change were common challenges, which impacted the navigation, design and implementation of some improvement projects. Some CIP009 fellows reflected that it was difficult readjusting their thinking to the CIP009 framework to avoid jumping to solutions, and coaches noted that the expectation of fellows to immediately generate solutions was challenging. Implementing projects and achieving behaviour change in a short timeframe was demanding, and depending on the project, required ongoing continuous support from coaches for an extended period of time to achieve desired outcomes. Implementation of projects was challenging, both in gaining stakeholder buy in and engagement and adoption of protocols, to achieve practice change and translation of evidence into practice. Several teams had not integrated consumer codesign into their improvement planning and design, and noted that this was an oversight, acknowledging the importance of consumer input as something that they would improve upon in future projects.

*“The teams that would be*,* um*,* overseeing those patients are quite resistant to change. They probably have quite a lot of change fatigue*,* and so when [our change initiative] was originally put through the senior consultants*,* they were like*,* ‘Absolutely not. No way’. So*,* there’s potential that you may come up with an option for*,* you know*,* an alternative pathway and alternative location. But the barrier then may be*,* ‘No*,* we don’t want to change anything. Let’s just leave it as it is’. So*,* it may be a very long-term solution that may take a lot of discussions and a lot of ongoing*,* and you know*,* mitigation strategies to say*,* ‘Oh*,* OK*,* the reason it would be a better option for patients is because we’ve engaged with consumers*,* and this is their feedback. This is a safety mechanism’.” (p7*,* CIP009 coach)*

#### Theme 7: CIP009 training and support shortcomings

##### Training shortcomings

The length of the CIP009 3.5-day training sessions was perceived as too long for some staff to be away from clinical duties, with some staff feeling burdened if their roles were not backfilled.

*“We had so many conflicting demands. And so*,* like*,* my phone was going constantly*,* you know, we had no cover. No one was covering our roles like so*,* taking three days off our normal jobs*,* it just meant that when we got back*,* we were swamped with so much work.” (p12*,* CIP009 fellow)*

Some CIP009 fellows felt that lectures were too long, with some repetitive, redundant, superficial and disjointed content, and felt that the guest lectures were not given enough context to be relevant. Some team members were observed to not stay for the whole duration of the training days, supporting these concerns. Several CIP009 fellows felt that there was not enough group planning time with their team to progress their project, perhaps resulting in a missed opportunity to maximise momentum and enthusiasm from the training days.

*“I think only a small amount of that [CIP training] time is dedicated to actually working on the actual problem. Like*,* you do little bits of it*,* but I wonder*,* if the teams*,* given they are actually together and the time’s already secured*,* would benefit from 1/2 day at the end [of the training session] or something to*,* um*,* really get the [project] kick started.” (p19*,* CIP009 fellow)*

Similarly, some CIP009 fellows felt that they had limited *team time* with their coach during the training days, particularly when coaches were split across multiple teams, leaving some teams unsure how to proceed while waiting for their coach to return. Several fellows noted that CIP009 projects were outcome focused rather than capability focused as past CIPs have been, with additional wrap around support from coaches meaning that the team members had fewer opportunities to practice the skills learnt in the CIP009 training course.

##### Communication issues

Communication about expectations of commitment was another challenge identified. Some fellows felt presentation fatigue after presenting project results across multiple forums (the midpoint session, graduation day and to executive committees), suggesting they could record their presentations to reduce time away from clinical duties. CIP009 fellows also noted that the lack of notice around the commencement of the CIP009 program and training days created scheduling conflicts with clinical commitments, increasing staff burden. As a result of limited communication, some fellows felt they were being enrolled in the program as a result of poor performance and had negative reactions to being nominated by Divisional Directors and Heads of Departments. That quickly dissipated once they understood the purpose of the program and why their role was integral to the improvement project. Some felt the prescriptive nature of this process reduced their internal motivation, while others felt that such external support for the projects was motivating.

##### Outcomes focus limiting codesign

Many CIP009 fellows felt that the rapid design and top-down selection of project problems by executive, rather than by each team impacted their engagement with the project initially, and limited opportunities for codesign with project team members. This resulted in some topics being seen as less valuable or meaningful to solve compared to others.

*“This year*,* because it was like that focus on ramping and we got allocated our thing*,* it did*,* it wasn’t the priority for me… I would have chosen a different priority.” (p29*,* CIP009 fellow)*

##### Scepticism related to complexity of issues

There was some scepticism noted about whether the CIP framework and 12 CIP009 projects would be able to impact patient flow and ramping in a significant way, with the sentiment that the CIP009 framework was useful for some projects, but not all. These participants highlighted that CIP was one of several methodologies being supported by the LHN working towards enhancing patient flow.

*“It was a little bit shallow*,* in that it was maybe asking for such a huge problem like ramping*,* you’ve got to delve way deeper than the CIP course did…So*,* [CIP’s] really good for little problems*,* I think. Like*,* really good for some money saving*,* streamlining little problems that you would have on the wards or in outpatients or wherever.” (p24*,* CIP009 fellow)*

##### Sustainability planning issues

In terms of sustainability, several CIP009 fellows discussed how they had not yet set plans in place for ongoing monitoring and adjustment of their projects. This may be reflective of the stage the teams were at, still focused on problem clarification, solution generation and implementation at the time of interviews. There was, however, concern that projects would *drop off the radar* once Faculty coaching support was reduced, and competing priorities took over fellows’ workloads, particularly for projects viewed as person dependent.

The seven themes and subthemes representing determinants for CIP009 were deductively mapped against the five domains of the CFIR framework (Innovation, outer setting, inner setting, individual and implementation process) [28] (Table 3). Mapping these strengths and challenges against the theoretical framework reinforced how each subtheme was aligned with the different levels of determinants most likely to influence the implementation of CIP009 and the 12 CQI interventions. A large proportion of key strengths and challenges were mapped to the inner setting domain of the intervention relating to teams and culture, highlighting the importance of awareness of CIP, multidisciplinary teamwork and cohesion, engagement with stakeholders, a lack of hierarchy, and accountability. The innovation domain largely highlighted strengths of the CIP009 including training content, the support from the skilled internal Faculty while the outer setting domain largely included challenges like access to data and workforce capacity. The individual domain reflected strengths such as the internal motivation of CIP009 fellows to drive projects and improve workflow across the organisation, while the implementation domain reflected the progress of the CIP009 projects, having not yet reached stage of project sustainability planning.

**Table 3:**
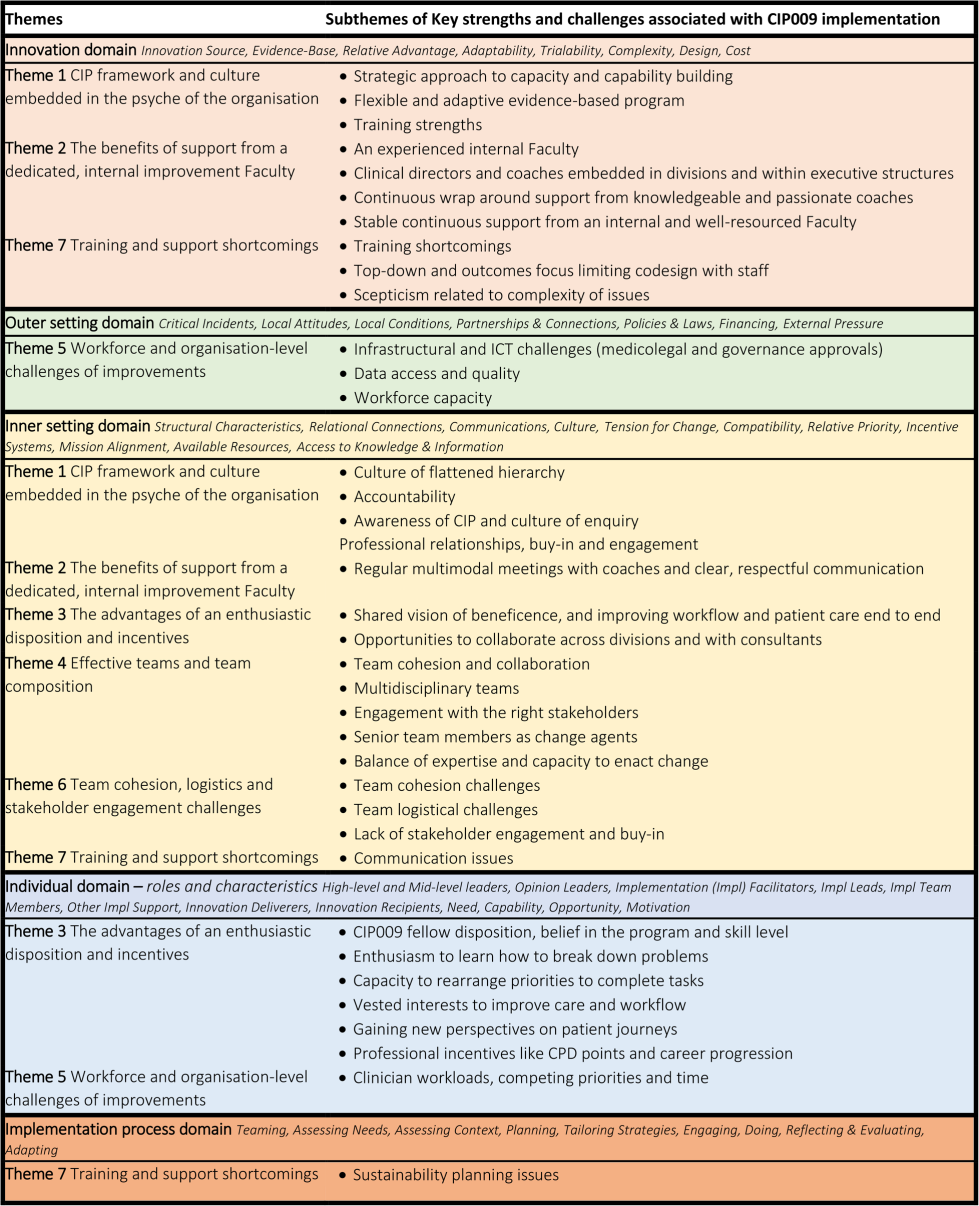
Strengths and challenges of CIP009 mapped against the CFIR domains [28]

**Fig. 1:**
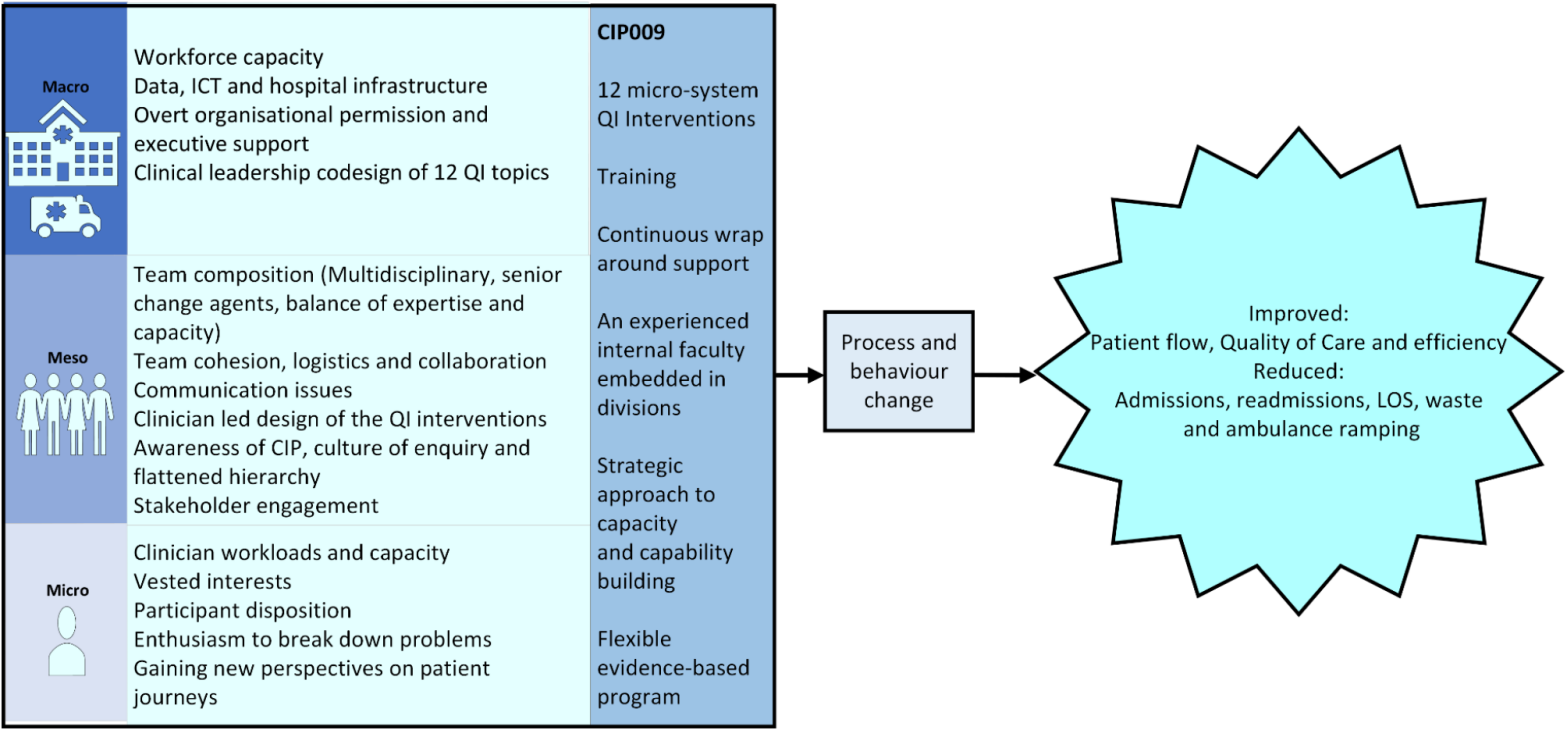
Key attributes of the SALHN CIP009 program

The key subthemes of the CIP009 were then collapsed into a more simplified structure of macro (hospital, outer setting), meso (teams, inner setting) and micro (individual) levels of the SALHN organisation, along with the key elements of the CIP009 program such as training and wraparound support from the Faculty. The fundamental elements of the CIP009 that were perceived to contribute to the implementation of CIP009 and its organisation-wide goal of improved patient flow and reduced ramping can be visualised in Fig. 1.

## Discussion

### Overview of the CIP009 evaluation

This evaluation of the SALHN CIP009, which encompassed interviews, focus groups, observations and document review, has identified key factors impacting the perceived success of the CIP009 improvement program across seven themes: The first four themes related to key strengths of CIP009, and the final three themes related to challenges.

### The learning health system

Upon reflection on the findings, it became apparent that the key elements of CIP009 described in this evaluation together contribute to a culture of continuous improvement to enhance the delivery of patient care. A concept of a *Learning Health System* has been rapidly evolving in recent years and refers to a systems approach to support organisations to establish data-informed continuous learning processes to incorporate best practice into routine care [34, 37, 38]. The Institute of Medicine defined an LHS as one where “*science*,* informatics*,* patient-clinician partnerships*,* incentives*,* and culture are aligned to promote and enable continuous and real-time improvement in both the effectiveness and efficiency of care*” ([39], p17).

This evaluation identified that CIP009 is underpinned by elements essential to a sustainable Learning Health System (LHS) [40]. For example, key LHS elements that were found in this evaluation of the SALHN CIP009 include improvements to health and care processes that are delivered through data-driven research that inform changes to practice [37, 39]. Similarly, continuous improvement cycles that utilise data and data infrastructure [41, 42] to inform practice change, followed by the implementation, assessment and amendments of the practice improvements [37, 43] were utilised by CIP009. Sustainable LHSs are grounded in systematic frameworks, have strong commitment from leadership to capture organisational priorities [43] and align incentives [41], are well resourced [42], and establish a supportive culture of continuous learning [41–43]. LHSs must be supported by an engaged and skilled workforce [42] with improvement capacity and capability [43]. An LHS can also enhance cross organisational collaboration by connecting siloed clinicians [43] as well as consumers and the community who are actively involved in the processes of continuous improvement [34, 41].

CIP009 has contributed to the development of these LHS elements within SALHN, with many of the themes from this evaluation reflected in the LHS literature. CIP009 has demonstrated the importance, and indeed the challenges, of access to quality routine service delivery data to inform the design of interventions at the patient-clinician interface (Theme 5) [43]. The longevity of the program has enabled CIP to evolve, establishing a systematic framework, CQI infrastructure, and a Faculty of knowledgeable personnel to provide continuous support and facilitate change (Theme 1) [43].

The novel combined top-down and bottom-up nature of CIP009 resulted in executive support for and investment in the program, while retaining CIP009 fellow design and ownership of the projects [44], and motivation to sustain changes (Themes 1 and 2) [45]. Strong leadership support was perceived to contribute to the uptake and adoption of CIP009 [46]. This support combined with the sustained resourcing for CIP009 has helped to build capability within the workforce (Theme 2) [43], implement CQI interventions [47], and enhance team accountability (Themes 1 and 2) [23]. CIP009 projects were also closely aligned with organisational priorities achieved through leadership codesign of CQI project topics to improve patient flow (Theme 1) [48]. CIP009 established a culture of inquiry and continuous learning [43, 49], with inhouse continuous wrap-around support [47] to develop technical skills and CQI knowledge (Themes 1 and 2) [16]. The perceived cultural change at the organisational level (Theme 1) was achieved through increased awareness and engagement with the structured framework, language and methodology [6], potentially mitigating loss of CIP knowledge from staff turnover [44].

CIP009 also focused on engagement and co-design of CQI interventions with key stakeholders (Theme 1). Stakeholder and leadership buy-in was facilitated through a combination of a flattened hierarchy and encouragement of equal participation by team members (Theme 1) [50, 51], and continuous support from coaches (Theme 2) [52, 53]. The transformational leadership style [54] used by coaches ensured momentum and coordination was maintained, and change mechanisms effectively communicated to persuade change adoption (Theme 2) [47]. CIP009 fellow belief in the value of reducing unwarranted variation in practice and vested interests to improve care and workflow (Theme 3) [55], multidisciplinary and interprofessional teams who provided insight into systems and processes [16] and interdivisional collaboration (Theme 4) [43] also contributed to staff buy-in. These elements are each fundamental to address the wicked problems that persist within the complex adaptive system that is healthcare [56]. The ongoing nature of CIP has meant that a large proportion of SALHN staff have graduated from CIP training, developing a community of CQI experts (Theme 1) [16]. CIP009 has endeavored to embed best practice into routine care [23], and improve the value and efficiency of processes [40] through data driven improvements [43], contributing to the establishment of an LHS within SALHN.

### Quality improvement and implementation science

CIP009 teams faced implementation barriers such as overcoming resistance to change and achieving buy-in, in particular with the development and adoption of protocols to reduce unwarranted variation (Theme 6), both common barriers to guideline adherence [57, 58]. This speaks to an aim to enhance translation of evidence into practice [58], the foundation of Implementation science [59], while concurrently aiming to improve efficiency and effectiveness of processes and practice [60]. Implementation science elements that focus on the diffusion, dissemination, implementation, adoption and sustainability of the CQI interventions could be further integrated within the initial stages of the CIP project planning framework, to provide opportunity to identify, plan for and mitigate implementation challenges [61].

Implementation science highlights the importance of change efforts being grounded in principles of behaviour change [60]. Guidance from behaviour change models such as the Theoretical Domains Framework (TDF) [62] during the CIP009 project planning phase, may increase the likelihood that interventions will achieve change [62]. To ensure changes are effectively embedded within organisational practice and sustained, long-term periodic feedback and evaluation of interventions should also be embedded within the early CQI project planning phase, to ensure the intervention remains applicable to the setting and sustainability is considered from the beginning of the project [59, 63, 64].

Further integration of implementation science and CQI theories and strategies would guide CIP fellows on how to best support change adoption by considering local contexts and determinants (barriers and facilitators) of change, as outlined in the CFIR [28] and Table 3, and to discern whether their change initiatives have been maintained, sustained and improved over time [59]. In line with this, the nature of support from an internal CIP009 Faculty enabled coaches to provide contextually relevant guidance and project facilitation.

Robust planning for implementation, sustainability and accountability, informed by an evidence based framework such as the Exploration, Preparation, Implementation, Sustainment (EPIS) Framework [65], the Reach, Effectiveness, Adoption, Implementation, and Maintenance (RE-AIM framework) [66] or the Proctor Taxonomy of Implementation Outcomes [67] would ensure the best opportunities for the implemented changes to continue [34]. The concept of sustainability is already incorporated within the SALHN CIP009 Continuous Improvement framework. However, the limited planning for, or application of sustainability processes reported by CIP009 participants both reflects their early stage of progress within the SALHN 8-step framework but also indicates an opportunity for sustainability planning to be integrated at an earlier stage of CIP009. Quality improvement and implementation science differ methodologically, however there is potential for synergies that could enhance CIP patient care improvements. The bottom-up and top-down nature of this CQI program engages local stakeholders with strong leadership support and continuous measurement and adaptation to practice changes. This may be complemented by implementation science insights into mechanisms for contextually specific practice and behaviour change underpinned by theory and evidence. Systematic incorporation of implementation science frameworks may promote planning for both summative outcomes assessment as well as interim progress assessments to support adaptations and project sustainability [34].

### Opportunities for improvement

Reflecting on the key perceived challenges of CIP009, overcoming limited clinician time to engage in CQI projects (Theme 5) is essential to establish an effective Learning Health System, and requires further organisational commitment to protect and resource clinician time for CQI involvement [43]. Multimodal and online modules of training may enhance the accessibility of CIP resources [16, 68]. Similarly, CIP resources could be provided in an electronic format, within a repository of trusted and endorsed CQI education, support, and data analysis training resources, CIP case studies, online lectures to enable fellows to refresh their understanding of concepts, and additional data analysis resources for those fellows who want to extend their learning. A blended virtual and face-to-face model, along with greater protected time for training and implementation of the projects, may support those clinicians with competing clinical priorities (Themes 5 and 7) [69]. In saying this, it is worth noting that the face-to-face element of the training had perceived benefits of increased networking and collaboration with clinical members, and thus the provision of electronic training resources may introduce a trade-off of reduced engagement in the course. If, however, a blended model enables ongoing access to training resources, it is likely to facilitate further engagement in the program [70]. To increase the efficiency of training days and the amount of dedicated coach-team time (Theme 7), training days could be split into two parallel cohorts with practical workshops running concurrently to theory-based lectures. This would enable team time with coaches to be staggered; while one cohort listens to lectures, the other could engage in practical project planning activities with Faculty staff.

Workforce and organisational challenges, such as limited data access and quality [7] need to be addressed to achieve successful CQI implementation and an effective LHS [34], specifically to enhance capacity to design locally appropriate data-informed improvement projects (Theme 5) [43]. Both increased and timely access to electronic medical record data and improved quality of data will contribute to the developing LHS supported by CIP009 [37]. Future improvement projects will also be strengthened by increased consumer partnership and codesign of projects to improve healthcare service delivery [71]. These partnerships may be informed by the *Building successful partnerships in healthcare QI: A capability development framework for service users*,* families*,* communities*,* and staff* [71]. The top-down nature of project topic selection resulted in variable responses from CIP009 fellows. Involving clinicians at an earlier stage of the topic selection process, through a brief survey, may ensure projects are clearly aligned with perceived need from both executive and clinician stakeholders.

### Strengths and limitations

Strengths of the study include the use of member checking, use of multiple coders, as well as triangulation of data across three cohorts, and across three methods of data collection (interviews, focus groups, observations, and document review) to enhance the trustworthiness of the data [72]. There may have been self-selection bias [73] in recruitment, as those participants who chose to engage in an interview or focus group may not represent the cohort of CIP009 fellows. Not all CIP009 teams were interviewed or observed, which reduces how generalisable the findings are across the 12 teams. Due to the complexity of the 12 CIP009 projects, and the corresponding extension of the program, teams were typically still in the early stages of the SALHN 8 step framework when data collection was conducted, meaning teams hadn’t fully implemented their projects nor assessed their impact. This evaluation therefore lacked data about the challenges and strengths experienced during the implementation stage of the individual quality improvement projects, as well as the ongoing accountability and sustainability of the improvement projects.

## Conclusion

In conclusion, the 12 CIP009 clinical micro-system interventions together aimed to contribute to a common organisational goal of reduced ambulance ramping by increasing patient flow, and reducing admissions, readmissions, length of stay and unwarranted clinical variation. Protocolisation of practice change was a common tool used to enhance the delivery of evidence-based practice to patients. The continuous wrap around support, multidisciplinary collaboration, culture of enquiry and structured framework of CIP009, as well as the top-down support in combination with bottom-up intervention design, has resulted in a CQI training program that is perceived to effectively develop staff skills and facilitate progress of micro-system improvements to achieve macro-outcomes. Incorporation of implementation science principles within the continuous improvement framework may further support the implementation and sustainability of future CIP projects.

## Data Availability

Interview transcripts are not publicly available to protect the confidentiality of study participants. However, data such as codes, and anonymised quotes may be available from the corresponding author (PH) upon reasonable request.

## Appendix

**Table 4:** Semi-structured interview topic guide

Q	Example interview questions and prompts
1	***What is your CIP 009 role****(executive*,* facilitator*,* project team member)? What is your Clinical level*,* specialty and area of expertise? How long have you been clinically practicing?*
*2a*	**Describe what your role in the CIP 009 program/ project team involves?** H*as your role changed since previous iterations of the CIP program?*
2b	**Which skills/ experience were needed to deliver and support the CIP 009 program / design and implement the CIP project?**
2c	**How do you feel about the additional workload associated with the CIP 009 project?***Positive /negative impact*,* overburdened/stressed?*
3a	**How CIP 009 project topics selected?***What were the criteria? (Department needs-based? informed by health round table/ conversations*,* specific to the LOS reduction target or is it more widely applicable to other targets such as safety?*)
3b	**How were team members selected for each project?** *What were the criteria?*
3c	**What CIP training was provided to you and how useful and informative were the CIP training**,** presentations**,** support**,** and CIP materials?***Did the training adequately equip you to conduct the CIP CI project?*
3e	**What are the strengths and weaknesses of the CIP 009 program?** *Do you see the CIP CI as an advantage to your organisation? How do you feel about the CIP CI intervention being used in your setting? How complicated is the CIP CI program?*
3 g	**What kind of information do you collect as you implemented the CIP project?** *How do you assess progress towards goals? Have you received feedback about the progress of the project?*
3 h	**Has the planning / implementation process been straightforward**,** and was implementation as you intended?***How have you prioritised project goals?*
4a	**What adaptations to the CIP methodology did you make/observe project teams making? What support did you provide in that instance?**
*5a*	**What were the challenges/barriers that you /the teams faced during CIP training**,** and the design and implementation of the project? Any surprises?** *Prompts: training*,* support*,* team/topic choice*,* design process/setting the problem/aim/diagnostics/outcomes/processes/data collection*,* implementation within departments*,* project acceptability*,* perceptions of influential stakeholders*,* logistics of team meetings*,* physical spaces*,* resources*,* time*,* staffing*,* Fit with the values of the org.*,* Fit with workflow processes/practices*,* self confidence that you can implement the project*
5b	**How did you /the team make decisions to overcome CIP project challenges?**
*5c*	**What facilitated the engagement with CIP / design and implementation of the CIP projects?** *Any surprises?* *Prompts: Professional development/ research activity benefits in relation to professional memberships*,* a need for change*,* perceived Importance of project*,* incentives (financial or other)*,* confidence that you could successfully implement the project*, champions of the project/opinion leaders
5d	**How does the CIP fit in with expectations and policies from SALHN and SA health?** *Is the improvement methodology supported by SA health? Do they provide facilitation (incentives) or barriers to undertake this work?*
6a	**Which supports from CIP facilitators/executives do project teams most need to effectively design and implement their project?***What support did you expect and how did that compare to what you received (from facilitators*,* execs*,* other project members)? Were the supports adequate? Prompts (processes*,* procedures*,* people*,* IT support)*
6b	**Did projects receive support/endorsement from departments/hospital?***What kinds of support where you expecting from the hospital executives (resources*,* staffing*,* time to conduct the CIP project? How much support did you receive? What was your method of resolution if you didn’t receive the support needed from the hospital to implement the project?*
7	**What plans are in place to ensure project evaluation and sustainability is ongoing?** *What support do you need to sustain your project?*
8	**Do you have any suggestions for the way the CIP program could be designed/run/organised/delivered differently to work more effectively in your setting?** Are there procedures or ways of working that would make it easier to implement the CIP CI program and Projects?
9	**Is there anything else that is important that we haven’t yet covered?**

## Abbreviations

COREQ: Consolidated Criteria for Reporting Qualitative Studies
CFIR: Consolidated Framework for Implementation Research
CPD: Continuing Professional Development
CIP009: Continuous Improvement Program 009
The Faculty: Continuous Improvement Unit (CIU)
CQI: Continuous Quality Improvement
ED: Emergency Department
EPIS: Exploration, Preparation, Implementation, Sustainment Framework
HREC: Human Research Ethics Committee
LHS: Learning Health System
LNR: Low and Negligible Research
LOS: Length of Stay
SALHN: Southern Adelaide Local Health Network
TDF: Theoretical Domains Framework
